# Рибосомное профилирование как инструмент исследования трансляции у растений: основные итоги, проблемы и перспективы

**DOI:** 10.18699/VJ21.028

**Published:** 2021-05

**Authors:** D.A. Afonnikov, O.I. Sinitsyna, T.S. Golubeva, N.A. Shmakov, A.V. Kochetov

**Affiliations:** Institute of Cytology and Genetics of the Siberian Branch of the Russian Academy of Sciences, Novosibirsk, Russia Novosibirsk State University, Novosibirsk, Russia; Institute of Cytology and Genetics of the Siberian Branch of the Russian Academy of Sciences, Novosibirsk, Russia Novosibirsk State University, Novosibirsk, Russia; Institute of Cytology and Genetics of the Siberian Branch of the Russian Academy of Sciences, Novosibirsk, Russia Novosibirsk State University, Novosibirsk, Russia; Institute of Cytology and Genetics of the Siberian Branch of the Russian Academy of Sciences, Novosibirsk, Russia Novosibirsk State University, Novosibirsk, Russia; Institute of Cytology and Genetics of the Siberian Branch of the Russian Academy of Sciences, Novosibirsk, Russia Novosibirsk State University, Novosibirsk, Russia

**Keywords:** ribosome profiling, Ribo-seq, RNA-seq, translation, plants, abiotic stress, biotic stress, рибосомное профилирование, Ribo-seq, RNA-seq, трансляция, растения, абиотический стресс, биотический стресс

## Abstract

Экспрессию эукариотических генов можно регулировать на нескольких этапах, включая трансляцию мРНК. Известно, что структура мРНК способна влиять как на эффективность взаимодействия с аппаратом трансляции в целом, так и на выбор сайтов инициации трансляции. Для исследования транслируемой фракции транскриптома были разработаны экспериментальные методы анализа, наиболее информативным из которых является рибосомное профилирование (РП, Ribo-seq). Первоначально созданный для использования в дрожжевых системах, этот метод был адаптирован для трансляционных исследований на многих видах растений. Технология включает выделение полисомной фракции и высокопроизводительное секвенирование пула сегментов мРНК, связанных с рибосомами. Сравнение результатов покрытия транскриптома прочтениями, полученными по протоколу рибосомного профилирования, с аналогичным результатами по секвенированию транскриптома дает возможность оценить эффективность трансляции для каждого транскрипта. Точные положения рибосом, определенные на последовательностях мРНК, позволяют определять трансляцию открытых рамок считывания и переключение между трансляцией нескольких рамок считывания – феномен, при котором с одной матрицы РНК происходят считывание двух или более перекрывающихся рамок и биосинтез разных белков. Преимущество метода заключается в том, что он дает возможность получить количественные оценки покрытия рибосомами мРНК и может выявлять относительно редкие события трансляции. Использование этой технологии позволило классифицировать гены растений по типу регуляции их экспрессии на уровне транскрипции, трансляции или на обоих уровнях. Обнаружены особенности структуры мРНК, которые влияют на уровни трансляции: формирование квадруплексов G2 и наличие специфических мотивов в области 5’-UTR, GC-состав, наличие альтернативных стартов трансляции, влияние uORF на трансляцию нижестоящих mORF. Показано, что изменения регуляции экспрессии генов на уровне трансляции возникают в ответ на биотический и абиотический стрессы, а также в процессе развития растений. В обзоре кратко рассмотрены методология РП и перспективы ее применения для исследования структурно-функциональной организации и регуляции экспрессии генов растений.

## Abstract (en)

The expression of eukaryotic genes can be regulated at several stages, including the translation of mRNA. It is known that the structure of mRNA can affect both the efficiency of interaction with the translation apparatus in general and the choice of translation initiation sites. To study the translated fraction of the transcriptome, experimental methods of analysis were developed, the most informative of which is ribosomal profiling (RP, Ribo-seq). Originally developed for use in yeast systems, this method has been adapted for research in translation mechanisms in many plant species. This technology includes the isolation of the polysomal fraction and high-performance sequencing of a pool of mRNA fragments associated with ribosomes. Comparing the results of transcript coverage with reads obtained using the ribosome profiling with the transcriptional efficiency of genes allows the translation efficiency to be evaluated for each transcript. The exact positions of ribosomes determined on mRNA sequences allow determining the translation of open reading frames and switching between the translation of several reading frames – a phenomenon in which two or more overlapping frames are read from one mRNA and different proteins are synthesized. The advantage of this method is that it provides quantitative estimates of ribosome coverage of mRNA and can detect relatively rare translation events. Using this technology, it was possible to identify and classify plant genes by the type of regulation of their expression at the transcription, translation, or both levels. Features of the mRNA structure that affect translation levels have been revealed: the formation of G2 quadruplexes and the presence of specific motifs in the 5’-UTR region, GC content, the presence of alternative translation starts, and the influence of uORFs on the translation of downstream mORFs. In this review, we briefly reviewed the RP methodology and the prospects for its application to study the structural and functional organization and regulation of plant gene expression.

## Введение

Разработка и использование современных масштабных и высокопроизводительных геномных технологий привели к радикальному изменению инструментария проведения молекулярно-биологических экспериментов за последние несколько десятилетий. Так, исследование транскриптомного профиля в масштабах генома (RNA-seq) использовано для идентификации генов, экспрессия кото-рых изменяется в ответ на сигналы окружающей среды и развития у большинства, если не у всех, сельскохозяйственных растений, а также у патогенных микробов (Kazan, Gardiner, 2018; Lanver et al., 2018; Baek et al., 2019; Zumaquero et al., 2019; Kang et al., 2020). Такие исследования обычно выявляют тысячи генов, которые при определенных условиях экспрессируются по-разному. Группировка дифференциально экспрессирующихся генов по ключевым функциональным категориям или онтологиям генов (GO) обеспечивает идентификацию главных клеточных процессов, лежащих в основе развития растений и их ответа на стресс. Однако становится все более очевидным, что мРНК разных генов не одинаковы по эффективности и специфичности трансляции и связь между количеством транскрипта и количеством синтезируемого с него белка может носить сложный характер, поскольку на экспрессию продукта гена оказывает влияние множество факторов, существенная доля которых связана с процессами трансляции мРНК (Lei et al., 2015; Merchante et al., 2015). Таким образом, хотя анализ транскриптома представляет собой необходимый этап при изучении паттернов экспрессии генов и механизмов генетического контроля различных процессов, он не является достаточным. Поэтому первостепенное значение для понимания того, какие продукты гена и в каком количестве образуются в результате его экспрессии, имеет исследование других процессов, включающих стабильность мРНК, особенности ее трансляции, стабильность полипептида, возможность посттрансляционных модификаций и т.д

Для экспериментального изучения этого процесса в последние годы было разработано несколько высокопроизводительных подходов, например профилирование рибосом или рибо-секвенирование (далее РП, в литературе – Ribo-seq). Они дают возможность в масштабе всего транскриптома определять различные особенности процесса трансляции и оценивать их интенсивность на количественном уровне. В этой статье мы привели краткий обзор технологии РП, несколько примеров применения метода для исследования генов растений, которые позволили расширить представления о процессах, влияющих на развитие растений, а также на устойчивость к биотическим и абиотическим стрессам.

## Рибосомное профилирование как инструмент исследования трансляции

Точный мониторинг процесса трансляции был технически невозможен до тех пор, когда о методологии Ribo-seq впервые сообщили в 2009 г. (Ingolia et al., 2009). Этот метод позволяет получить «отпечаток» всех транслируемых мРНК (транслатом) с помощью транскриптомной идентификации коротких (~30 нт) фрагментов мРНК, физически связанных с рибосомами (см. рисунок, а). Метод достаточно прост и основан на выделении полисом (т. е. транслируемой фракции мРНК), обработке РНКазой 80S рибосом с находящимся внутри сегментом мРНК, выделении пула этих сегментов и их секвенировании. Далее биоинформатический анализ позволяет определить количество таких сегментов для различных мРНК в транскриптоме, что дает важную информацию об эффективности трансляции матриц – если таких сегментов мало, то уровень синтеза белка будет низким даже при большом количестве мРНК. Не менее важно то, что позиционирование защищенных рибосомами сегментов при их выравнивании на нуклеотидную последовательность мРНК дает возможность определить сайты инициации трансляции, т. е. транслируемые открытые рамки считывания.

**Fig. 1.**
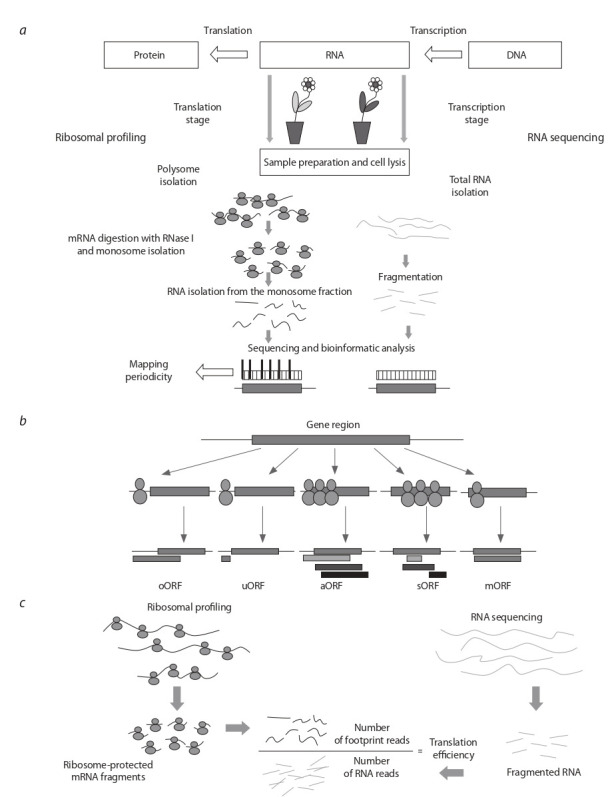
Schematic presentation of the ribosomal profiling technology in plants (а) Plant tissue is homogenized, the cells are lysed, and each sample is divided into two portions. The polysomal fraction is extracted from the first portion of the sample, the RNA is digested with RNase I to form mRNA fragments associated with individual ribosomes (monosomes) for the ribosome fingerprint method (”ribosomal footprint“). RNA is isolated from the monosomal fractions and used for library preparation and subsequent high-coverage sequencing of ribosome-bound mRNA fragments to determine the rate of instantaneous protein synthesis. The other portion of the sample is used to isolate total RNA to obtain a transcriptional profile in addition to ribosomal profiling. (b) Ribosomal profiling provides experimental identification of intensely translated transcript regions and identification of new translated open reading frames (ORFs), such as upstream ORFs (uORF), overlapping ORFs (oORF), alternative ORFs (aORF), small ORFs (sORF), in addition to main ORFs (mORFs). (c) Ribosomal profiling data not only provides information about the rate of instantaneous protein synthesis, but also allows determination of the relative translation efficiency of individual genes by comparing the reading intensity during sequencing of mRNA fragments of the ”ribosomal footprint“ with the number of specific transcripts

Первоначально разработанный для использования в дрожжевых системах (Ingolia et al., 2009), этот метод был адаптирован для трансляционных исследований на многих видах растений (Liu et al., 2013; Zoschke et al., 2013; Juntawong et al., 2014; Lei et al., 2015; Merchante et al., 2015; Hsu et al., 2016; Lukoszek et al., 2016). Протоколы Ribo-seq также были расширены для изучения процессов функционирования хлоропластных (Zoschke et al., 2013; Gawroński et al., 2018) и митохондриальных (Rooijers et al., 2013) рибосом.

В методе рибосомального профилирования ткани растений экстрагируют в буфере и обрабатывают РНКазой I. При гидролитическом расщеплении одноцепочечной мРНК остаются ее фрагменты, связанные с рибосомами. Фрагменты мРНК с рибосомами далее выделяют с помощью хроматографии или методом центрифугирования в ступенчатом градиенте сахарозы («сахарозная подушка»). Получается набор олигонуклеотидов характерной длины, ~30 нт, которая приблизительно соответствует размеру сайта посадки рибосомы. Последовательности произведенных таким образом олигонуклеотидов определяют на секвенаторах нового поколения с высоким покрытием. 

При сравнении результатов покрытия транскриптома прочтениями, полученными по протоколу рибосомного профилирования, с аналогичными данными, полученными для секвенирования транскриптома, можно оценить эффективность трансляции для каждого транскрипта. Такой анализ в масштабе всего транскриптома позволяет установить, как трансляция отдельных транскриптов соотносится между образцами. В основе этого метода лежит предположение, что все мРНК, связанные с рибосомой, подвергаются трансляции и, если скорость удлинения эквивалентна по всему транслатому, то средняя занятость рибосом является хорошим показателем скорости трансляции. 

Рибосомное профилирование имеет много преимуществ перед некоторыми другими методами профилирования трансляции (Jackson, Standart, 2015). Одно из них заключается в том, что локализация рибосом вдоль последовательности мРНК позволяет производить дополнительный контроль собранных данных. Например, участки, связанные с рибосомами, должны быть сконцентрированы в кодирующей части мРНК, отсутствовать в области 3ʹ-UTR и располагаться с периодичностью трех нуклеотидов, как следствие, кодонной структуры мРНК. Если в результате покрытия прочтениями библиотек Ribo-seq транскриптома такие особенности отсутствуют, то это может свидетельствовать о низком качестве полученных библиотек

Возможность получить расположение пиков покрытия рибосомами последовательностей мРНК на основе выравнивания прочтений Ribo-seq с разрешением на уровне кодонов сделала этот метод полезным для изучения механизмов и динамики трансляции. Точные положения рибосом, определенные на последовательностях мРНК, отображают периодичность кодонов. Это свойство позволяет точно определять трансляцию открытых рамок считывания (ORF) и переключение между трансляцией нескольких рамок считывания – феномен, при котором с одной матрицы РНК происходят считывание двух или более перекрывающихся ORF и биосинтез разных белков. Это, в свою очередь, помогает идентифицировать новые механизмы контроля трансляции, такие как события инициации трансляции в кодонах, отличных от кодона AUG, трансляции uORF, трансляции малых (sORF) и альтернативных ORF (aORF), которые ранее считались некодирующими или псевдогенами (Ingolia et al., 2009, 2011; Brar et al., 2012; Stern-Ginossar et al., 2012; Hsu et al., 2016). 

Другие примеры полезного использования точного позиционирования рибосом на мРНК – картирование сайтов начала трансляции с ингибиторами элонгации, наличие вышележащих открытых рамок считывания (uORF) и неканонических стартовых кодонов (Ingolia et al., 2011), более точное определение механизмов сканирования и инициации путем картирования малых рибосомных субъединиц 40S (Archer et al., 2016), а также действие специфических стрессоров или динамика рибосом после терминации (Andreev et al., 2017).

Дополнительная особенность метода – он позволяет получить количественные оценки покрытия рибосомами мРНК и может определять относительно редкие события трансляции (см. рисунок, б ). Среднее нормализованное (для длины ORF и глубины секвенирования) количество защищенных фрагментов рибосом (ribosome protected fragments, RPF), обнаруженное с помощью профилирования, обеспечивает оценку интенсивности синтеза белка (Ingolia et al., 2009). Кроме того, профилирование рибосом в сочетании с секвенированием РНК тех же образцов дает информацию об эффективности трансляции in vivo (см. рисунок, в), определяемой как скорость трансляции мРНК. Ее можно рассчитать, разделив среднюю плотность участков рибосомы данного гена на уровень экспрессии его мРНК, оцененный на основе анализа RNA-seq (Ingolia et al., 2009). 

Важной особенностью рибосомного профилирования также является то, что этот метод может быть адаптирован для изучения самых разных типов клеток или тканей любых организмов с очень небольшими модификациями из-за консервативности молекулярных и биофизических свойств рибосом, хотя для применения этой технологии у различных организмов может потребоваться техническая оптимизация (Brar, Weissman, 2015).

## Основные итоги применения технологии рибосомного профилирования у растений

Экспрессию генов во время адаптации к биотическим и абиотическим факторам стресса, а также в процессе развития интенсивно изучали на уровне транскриптомов растений и различных генов-кандидатов, которыми можно манипулировать для повышения устойчивости к стрессу. С появлением технологий рибосомного профилирования эти данные могут быть сопоставлены с результатами анализа профилей трансляции для оценки вклада различных молекулярных механизмов трансляции в регуляцию экспрессии генов.

**Трансляционная регуляция экспрессии генов в условиях абиотического стресса**

Абиотический стресс, такой как повышенные или пониженные температуры, или повышенная или пониженная интенсивность освещения, засуха, засоление или избыточное увлажнение почвы, оказывает значительное влияние на физиологическое состояние растений и, следовательно, на экспрессию их генов. Поэтому среди первых приложений метода РП были эксперименты по анализу реакции растений на абиотические стрессы. Метод РП был особенно полезен для понимания потенциальной роли, которую играют uORF в регуляции трансляции во время быстрых реакций на внешние стимулы, такие как кислородное голодание (гипоксия), тепловой стресс и водная депривация.

Ранее предполагалось, что uORF участвуют в регуляции генов в ответ на различные изменения окружающей среды (Hanfrey et al., 2002; Imai et al., 2006; Alatorre-Cobos et al., 2012). Однако из-за отсутствия экспериментальных данных оставалось неясным, транслируются ли uORF в белки. Профилирование рибосом позволяет идентифицировать uORF и другие регуляторные области, такие как квадруплексы G2 (третичные структуры ДНК, состоящие из тетрад гуанина). Анализ адаптивных ответов на тепловой стресс у Arabidopsis thaliana с помощью метода РП в сочетании с секвенированием РНК выявил, что экспрессия генов перепрограммируется во время длительного теплового стресса за счет преимущественной трансляции генов, содержащих квадруплексы G2 в их 5ʹ-UTR. Это было очевидно из корреляции между плотностью считывания RPF по структурам квадруплексов G2 и повышенными уровнями экспрессии нижележащих основных ORF (mORF) (Lukoszek et al., 2016).

Показано, что гипоксия оказывает крайне негативное влияние на эффективность трансляции у A. thaliana с примерно 100-кратным ее снижением для некоторых мРНК (Juntawong et al., 2014). Это в основном объясняется сни­жением инициации трансляции из-за уменьшения занятости рибосом в стартовых кодонах транскриптов генов, не чувствительных к гипоксии (Juntawong et al., 2014). Эти данные расширили понимание механизмов трансляции, подтвердив, что в нормальных условиях роста uORF снижают трансляцию многих mORF. Напротив, для небольшого числа проанализированных генов uORF не влияют на трансляцию mORF в условиях низкого содержания кислорода. P. Juntawong с коллегами (2014) установили, что «отпечатки рибосом» на мРНК после ее расщепления нуклеазой можно использовать для профилирования рибосом у растений. 

Ограничение количества влаги также приводит к глобальному изменению уровней экспрессии генов, регулируемых как на транскрипционном, так и на трансляционном уровне. L. Lei с коллегами (2015) использовали профилирование рибосом для выяснения регуляции трансляции экспрессии генов в ответ на засуху у кукурузы. Они установили, что кратные изменения транскрипции, вызванные засухой, умеренно коррелировали с трансляционными изменениями. В их работе показано, что 41 % всех генов, чувствительных к засухе (т. е. генов, экспрессия которых регулируется на уровне транскрипции, трансляции или на обоих уровнях), регулируется на уровне транскриптции/трансляции несогласованно. Это указывает на то, что в условиях засухи регуляция экспрессии происходит независимыми путями: для одних – на уровне транскрипции, для других – на уровне трансляции. Авторы также сообщили, что на эффективность трансляции влияют такие характеристики последовательности, как содержание нуклеотидов G, C, длина кодирующих последовательностей и нормализованная минимальная свободная энергия, определяющая стабильность последовательности вторичных структур (Lei et al., 2015).

Один из ключевых гормонов стрессового ответа растений – этилен. Путь передачи его сигналов пересекается с путями передачи сигналов других фитогормонов в процессе опосредованного ответа как на биотические (Schenk et al., 2000), так и на абиотические стрессы (Abeles et al., 2012). Исследование, в котором проведено сравнение этилен-индуцированных состояний транскриптома и транслятома у A. thaliana с использованием комбинированных подходов РНК-seq и Ribo-seq, показало, что зависимость между оценками количества транскриптов и количества их фрагментов, связанных с рибосомами, является слабой (коэффициент детерминации R2 = 0.22) (Merchante et al., 2015). В этой работе также продемонстрировано, что после воздействия этилена изменяются активация транскрипции и эффективность трансляции регуляторов передачи сигналов этилена, таких как EBF1 и EBF2, что указывает на ключевую роль транскрипционной и трансляционной регуляции экспрессии генов ответа на этилен. Изменение эффективности трансляции EBF1 в ответ на обработку этиленом опосредуется 3ʹ-UTR-областью гена EBF1 в присутствии функционального EIN2, что не требует присутствия комплекса EIN3/EIL1 или других ключевых транскрипционных факторов ответа на этилен, как при регуляции на уровне транскрипции (Merchante et al., 2015). В данном случае профилирование рибосом выявило ключевой компонент в регуляции передачи сигналов этилена, который был упущен из виду при использовании только транскриптомных подходов.

**Трансляционная регуляция экспрессии генов в условиях биотического стресса**

В процессе ответа растений на биотический стресс происходит транскрипционное репрограммирование большого количества генов (Schenk et al., 2000). Однако о трансляционном репрограммировании во время иммунного ответа растений было известно очень мало. G. Xu с коллегами (2017) выполнили глобальное рибосомное профилирование растений A. thaliana, обработанных полипептидом elf18, который содержит первые 18 аминокислот белка бактериального фактора элонгации Tu. Пептид elf18 – молекулярный паттерн, ассоциированный с патогенами (pathogen associated molecular pattern, PAMP), он идентифицируется при помощи паттерн-распознающих рецепторов растений. В результате этого запускается PAMP-активируемый иммунитет (pattern triggered immunity, PTI). Показано, что при таком ответе экспрессия ряда генов регулируется на трансляционном уровне. При этом для мРНК этих генов uORF могут оказывать как положительный, так и отрицательный эффект на трансляцию нижестоящих mORF. Эти исследователи обнаружили также богатый пуринами вышестоящий элемент, называемый R-мотивом, в 5ʹ-UTR-области генов с повышенной эффективностью трансляции после обработки растений elf18. Устранение репрессирующего эффекта R-мотива позволяет активировать гены иммунного ответа, поэтому R-мотив, по-видимому, важен для репрессии экспрессии генов в пути PTI у арабидопсиса (Xu et al., 2017). Эта работа подтвердила, что репрограммирование трансляции происходит на ранней стадии во время защитного ответа, скорее всего, до основных транскрипционных событий, и что в процессе активации PAMP-активируемого иммунитета транскрипционные и трансляционные изменения слабо коррелируют

Регуляция трансляции в процессе иммунного ответа, активируемого эффектором (effector triggered immunity, ETI), пока еще плохо изучена. Поэтому, чтобы прояснить детали этого механизма и сравнить особенности трансляционной регуляции растений в процессе иммунитета, активируемого эффектором и паттернами, H. Yoo с коллегами (2019) выполнили полногеномное профилирование рибосом в ответ на бактериальный патоген Pseudomonas syringae pv. maculicola, несущий эффекторный ген AvrRpt2, который кодирует белок, распознаваемый рецептором RPS2 у A. thaliana. Полученные данные демонстрируют, что в процессе паттерн-активируемого иммунного ответа регуляция трансляции четко скоординирована с ответом транскрипции. Отсутствие потенциальных мотивов консенсусной последовательности в нетранслируемых областях генов, меняющих свой экспрессию в ответ на стресс, предполагает, что скоординированное трансляционное изменение в процессе ETI происходит посредством модификаций трансляционного аппарата, а не через консенсусные последовательности в РНК. В исследовании также продемонстрировано, что регуляция трансляции в процессе ETI аналогична для различных иммунных рецепторов и что скоординированная регуляция генов, участвующих в нескольких метаболических путях, обеспечивает координацию метаболических изменений с иммунным ответом у растений (Yoo et al., 2019). Авторы показали, что, в распознавании AvrRpt2 и AvrRpm1 участвуют разные рецепторы хозяина (RPS2 и RPM1 соответственно), однако наборы дифференциально экспрессирующихся генов сигнальных путей, которые запускают эти рецепторы, существенно перекрываются. Так, общими для этих путей являются 50 % генов с повышенным уровнем транскрипции и 5 % – с пониженным. Что касается генов, у которых значимо меняется трансляционная активность, то их общая доля составляет выше 75 %.

**Трансляционная регуляция экспрессии в процессе развития растений**

Трансляционный контроль регуляции генов также важен для растений во время их развития для обеспечения синтеза онтогенетических и тканеспецифичных генных продуктов (Jiao, Meyerowitz, 2010; Mustroph, Bailey-Serres, 2010). Например, чтобы оценить трансляционную регуляцию экспрессии генов во время развития, опосредованного светом (фотоморфогенез) у A. thaliana, M.-J. Liu с коллегами (2013) использовали метод профилирования рибосом и картировали их положение в процессе трансляции на мРНК по всему геному в условиях света и темноты. Результаты показали, что трансляционная эффективность основных ORF была ниже в генах, содержащих транслируемые uORF, чем в генах без таковых. Авторы также сообщили, что у генов, являющихся мишенями миРНК, уровень трансляции существенно понижен в силу равномерного понижения покрытия рибосомами кодирующих последовательностей. Это исследование показало важную роль uORF и миРНК в регуляции трансляции в процессе фотоморфогенеза. 

Основываясь на этом исследовании, Y. Kurihara с коллегами (2018) применили подход к рибосомному профилированию у A. thaliana и продемонстрировали, что после воздействия синего света растения используют альтернативные стартовые сайты транскрипции, чтобы обойти опосредованное uORF ингибирование экспрессии генов. Это позволяет поддерживать экспрессию генов, регулируемую светом, на высоком уровне.

Метод рибосомного профилирования был применен недавно для сравнения эффективности трансляции в связи с изменениями уровней транскрипции у сои на разных стадиях развития семян (Shamimuzzaman, Vodkin, 2018). Авторы выявили, что эффективность трансляции у многих генов в процессе развития семян изменяется. Повышенная эффективность трансляции на отдельных стадиях развития семян была обнаружена для генов, связанных с развитием семян (например, генов протеаз, пептидаз и 2S альбуминов), что представляет уникальные особенности изменения регуляции трансляции в процессе развития (Shamimuzzaman, Vodkin, 2018). Эти результаты подчеркивают важность изучения регуляции экспрессии генов на уровне трансляции, для того чтобы установить, как развитие органов растений контролируется на молекулярном уровне.

**Обнаружение трансляционных событий из неаннотированных генов растений**

Несмотря на значительные усилия по расшифровке и анализу геномов растений, их последовательности содержат множество ORF, которые остаются неаннотированными. Для таких ORF неизвестно, являются ли они псевдогенами, продуцируют ли некодирующие РНК (нкРНК) или функциональные белки. Оказалось, что технологии рибосомального профилирования можно применять и для улучшения аннотации генома. В экспериментах по профилированию рибосом у A. thaliana модификация буферов, используемых для выделения фрагментов мРНК, связанных с рибосомами, позволила определить новые sORF, которые ранее считались нкРНК. Интересно, что многие из этих sORF эволюционно консервативны и содержат неканонические кодоны инициации трансляции, такие как CUG или ACG (Hsu et al., 2016). 

В исследовании (Wu et al., 2019) сборка транскриптомов на основе референсного генома и профилирование рибосом были использованы для улучшения аннотации генома томата (Solanum lycopersicum). Этот метод дал возможность идентифицировать сотни новых sORF из неаннотированных транскриптов, которые эволюционно консервативны. В данном исследовании также были сформированы транслированные последовательности для sORF, uORF из 5ʹ-UTR-областей генов, кодирующих белки. Верификация результатов, полученных с помощью рибосомального профилирования методами протеомики, показала, что некоторые из этих sORF продуцируют стабильные пептиды. Анализ обогащения аннотации этих транскриптов терминами генной онтологии выявил, что их функция связана с фосфорилированием/дефосфорилированием белков, сигнальными путями. Все это демонстрирует, что регуляция трансляции выполняет высокоуровневую регуляторную функцию в процессе жизнедеятельности клетки. Интересно, что H.-Y.L. Wu с коллегами (2019) обнаружили, что у томата экспрессия генов также регулируется глобально как на транскрипционном, так и на трансляционном уровне за счет микроРНК. Таким образом, применение метода профилирования рибосом оказывается ценным для подтверждения предсказания новых открытых рамок считывания и сайтов инициации транскриптов в масштабе всего генома (Willems et al., 2017).

Указанные выше исследования установили неканонические трансляционные события и позволили идентифицировать ранее не аннотированные малые белки, которые, вероятно, выполняют важные функции в растениях. Все это демонстрирует полезность экспериментов по рибосомному профилированию для более глубокой аннотации генов в геномах растений. 

**Проблемы и ограничения для применения методологии рибосомного профилирования у растений**

Несмотря на множество преимуществ, технология Riboseq в настоящее время сталкивается с некоторыми серьез-ными техническими трудностями. Среди них – необходимость устранения потенциальных загрязнителей и надежное выравнивание коротких прочтений с эталонным геномом или транскриптомами. В этом разделе мы кратко обсудим эти проблемы и предложим возможные пути их решения. 

Один из источников шума в результатах рибосомального профилирования – контаминация рибосомной РНК (рРНК) на стадии нуклеазной обработки. Она существенно затрудняет извлечение данных об информативных последовательностях, полученных в экспериментах по профилированию рибосом (Ingolia et al., 2009). Такая контаминация фрагментов мРНК фрагментами рРНК может быть устранена на основе специфической гибридизации или при помощи коммерческих наборов обеднения библиотек специфическими последовательностями РНК, а также и сочетанием обоих методов (McGlincy, Ingolia, 2017). С другой стороны, для предотвращения лигирования линкера с избытком 5.8S рРНК можно использовать маскирующие олигонуклеотиды (Faridani et al., 2016). Загрязнения рРНК можно устранить на этапе биоинформатической обработки, но этот подход требует получения большего покрытия при секвенировании.

Современные методы анализа транскриптомов, основанные на массовом секвенировании, обычно базируются на коротких прочтениях размером 100–150 п.н. для фрагментов с одиночным или парными концами (Wang et al., 2009), в то время как в более ранних подходах RNA-seq применяли прочтения длиной 25 и 32 п. н. (Marioni et al., 2008; Mortazavi et al., 2008). Так как секвенирование фрагментов, связанных с рибосомами, осуществляется короткими прочтениями, то это значительно увеличивает вероятность их выравнивания на множественные участки генома, что может приводить к возможным ошибкам в анализе результатов профилирования (Chhangawala et al., 2015). Кроме того, на уникальность выравнивания коротких прочтений более существенное влияние оказывают ошибки секвенирования.

Проблема множественного выравнивания прочтений в случае анализа транскриптомов растений усугубляется еще и тем, что большая часть существующих видов растений – полиплоиды (Wood et al., 2009). Например, у мягкой пшеницы (Triticum aestivum), аллогексаплоида, геном включает три субгенома, A, B и D, в которых всего ~100 000 генов. Около 85 % генома пшеницы – повторы, представленные многочисленными мобильными элементами (Ramírez-González et al., 2018). Во многих случаях последовательности генов гомеологов очень похожи, и чтобы получить выравнивание прочтений до совпадения, специфичного для гомеолога, требуется их большая длина (Pfeifer et al., 2014). Таким образом, изучение дифференциальной трансляции гомеологических генов затруднено, но может быть улучшено с помощью недавно разработанного подхода классификации субгеномов, при котором считывания сопоставляются с каждым субгеномом отдельно (Kuo et al., 2018). С другой стороны, проблема множественного картирования коротких прочтений может быть решена с помощью специальных инструментов, таких как алгоритм разрешения множественного картирования для фильтрации выравнивания (Kahles et al., 2016). 

Эксперименты по профилированию рибосом генерируют огромное количество данных, что создает значительную проблему для их анализа (Calviello et al., 2016). В дополнение к существующим инструментам для обработки последовательностей и выравнивания были созданы пакеты последующего анализа для идентификации трехнуклеотидной периодичности, дифференциальной трансляции и занятости кодонов: riboSeqR (Chung et al., 2015), riboWaltz (Lauria et al., 2018). На сегодняшний день разработаны два веб-сервера, RiboGalaxy (Michel et al., 2016) и riboviz (Carja et al., 2017), объединяющие набор инструментов, необходимых для анализа данных рибосомального профилирования, начиная с контроля качества исходной последовательности до финальной визуализации данных (Wang et al., 2017).

Дальнейшее изучение методов анализа данных рибосомального профилирования и подбор параметров выравнивания и покрытия для улучшения точности определения дифференциальной трансляции в значительной степени помогут при анализе и интерпретации результатов этих экспериментов. 

## Заключение

**Возможные перспективы методологии рибосомного профилирования у растений**

Большая часть современной информации о профилировании рибосом получена из нерастительных видов. Следовательно, проведение подобных исследований на растениях в различных условиях необходимо для обнаружения генов, экспрессия которых регулируется только трансляционно и до сих пор не выявлялась при анализе транскриптомов. Хотя профилирование рибосом – относительно новый метод, улучшения в этой технологии уже появляются. Так, этот метод можно использовать для изучения специфической трансляции органелл, кодируемой ядерными генами (Jan et al., 2014). В настоящее время все большее внимание привлекают методы анализа транскриптомов единичных клеток (Saliba et al., 2014). Они широко применяются в исследованиях на животных и имеют большой потенциал для понимания функции генов для единичных клеток в растениях (Efroni, Birnbaum, 2016). Сочетание этой новой методики с профилированием рибосом и такими подходами, как аффинная очистка транслирующих рибосом (Heiman et al., 2014), может иметь огромное значение при изучении регуляции трансляции, специфичной для определенного типа клеток (Mironova, Xu, 2019).

Дальнейшее улучшение определенных технических этапов экспериментов по профилированию рибосом могло бы способствовать более широкому внедрению этой техники. Например, в настоящее время подготовка библиотеки для глубокого секвенирования включает в себя несколько этапов лигирования и занимает много времени (обычно несколько дней). Кроме того, требование относительно больших количеств начальной РНК для подготовки библиотек может создавать проблемы. 

Таким образом, новый подход, называемый профилированием рибосом без лигирования, разработанный на мышах, может быть исследован на растениях, поскольку этот метод не требует лигирования, а создание библиотеки производится в течение дня с использованием всего лишь 1 нг РНК (Hornstein et al., 2016). Профилирование рибосом перспективно для идентификации rQTL по всему геному (рибосомный QTL), подобно тому как проводится анализ eQTL (экспрессионные QTL). 

## Conflict of interest

The authors declare no conflict of interest.
